# Long-Term Effects of Renal Artery Denervation

**DOI:** 10.3390/medicina57070662

**Published:** 2021-06-27

**Authors:** Vytautas Juknevičius, Andrius Berūkštis, Renata Juknevičienė, Eugenijus Jasiūnas, Pranas Šerpytis, Aleksandras Laucevičius

**Affiliations:** 1 Clinic of Heart and Vessel Diseases, Institute of Clinical Medicine at the Faculty of Medicine, Vilnius University, LT-03101 Vilnius, Lithuania; andrius.berukstis@santa.lt (A.B.); renata.ruseckaite@santa.lt (R.J.); Pranas.Serpytis@santa.lt (P.Š.); aleksandras.laucevicius@mf.vu.lt (A.L.); 2 Center of Informatics and Development, Vilnius University Hospital Santaros Clinics, LT-08661 Vilnius, Lithuania; eugenijus.jasiunas@santa.lt

**Keywords:** arterial hypertension, arterial stiffness, polypharmacy, renal artery denervation

## Abstract

*Background and Objectives*: Renal artery denervation (RDN) procedure is a broadly discussed method in the treatment of resistant hypertension. Many studies report short-term (3–12 months) results for blood pressure and arterial stiffness. The primary endpoints were changes in 24 h mean systolic blood pressure (BP) and office systolic BP 48 months after RDN. The secondary endpoints were changes in aortic pulse wave velocity and impact of polypharmacy on these variables. *Materials and Methods*: Renal artery denervation was performed in 73 patients treated for resistant hypertension; 49 patients remained in final analysis. Patient examination was carried out before the procedure, and subsequently at 3, 6, 12, 24, and 48 months later. Patients’ antihypertensive and overall medication regimens were carefully analysed. *Results*: Mean 24 h arterial blood pressure lowered and was sustained at lower levels for up to 48 months; median (interequartile range—IQR) from 158(23.5)/100(14.2) to 140(26.5)/86(16.2) mmHg. Mean reduction in 24 h ambulatory systolic BP was −11 ± 25 mmHg (95% CI, −20 to −2; *p* < 0.001), while office systolic BP reduced by −7 ± 23 mmHg (95%CI, −24 to −1; *p* < 0.02). A significant reduction in median aortic pulse wave velocity 12 months after the procedure (drop from baseline 11.2 [3.15] m/s (95%CI 6.1 to 16.2) to 9.8 [2.1] m/s (95%CI 6.1 to 13.7; *p* = 0.002)). After 48 months, there was no worsening compared to the baseline level of 10.3 [4.0] m/s (95% CI 6.9 to 17.8) (*p* > 0.05). The total mean number of antihypertensive drugs remained unchanged: 5.97(±1.1) vs. 5.24 (±1.45). A higher number of pills after 48 months was associated with higher aortic pulse wave velocity (1–5 pill group: 8.1 ± 1.6 m/s; 6–10 pill group: 10.9 ± 1.8 m/s; >11 pill group: 15.1 ± 2.6 m/s) (*p* = 0.003). *Conclusions*: Antihypertensive effect after renal denervation lasts up to 48 months with no worsening of arterial stiffness compared to baseline. In our study, polypharmacy was associated with increased arterial stiffness 48 months after the procedure.

## 1. Introduction

Arterial hypertension (HTN) remains the main risk factor for death and disability in the world [1]. Approximately 85% of hypertensive patients are aware of their disease and only about three-quarters take prescribed medications [2,3]. A meta-analysis of large-scale studies indicates a 12–15% prevalence of resistant hypertension (RH) among all patients suffering from arterial hypertension [4]. Resistant hypertension is defined by the American Heart Association/European Society of Hypertension/European Society of Cardiology (AHA/ESH/ESC) as elevated blood pressure (BP) remaining above goal despite the concurrent use of three or more antihypertensive medications of different classes, with one of the classes being a diuretic and all of the medications being prescribed at optimal dosage, or with controlled BP, but requiring four or more antihypertensive drugs [5]. Resistant hypertension (RH) is described as a clinical phenotype designating increased cardiovascular event risk. Moreover, RH is strongly linked with adverse outcomes such as coronary artery disease, heart failure, end stage renal disease, stroke and death compared to patients without RH and represents an important public health problem [2].

Over the past few years, there have been some studies with opposing conclusions on the effectiveness of renal artery denervation (RDN). SYMPLICITY HTN-1 and HTN-2 Trials presented a significant antihypertensive effect. Unfortunately, SYMPLICITY HTN-3 failed to prove a significant reduction in peripheral BP six months after RDN procedure in comparison to a placebo [6]. In 2017, SPYRAL OFF and ON trials established the BP lowering effect of RDN procedure compared to placebo. After the disappointing results from the SYMPLICITY HTN-3, the SPYRAL HTN-OFF MED studies were crucial in providing proof of the efficacy of RDN procedure without adding antihypertensive medications [7]. In addition, interim data from the SPYRAL HTN-ON MED involving patients treated with 2–3 antihypertensive medications seem promising [8]. While all of these trials share quite the same study protocol, the biggest difference between them is the number of antihypertensive medications.

Aortic pulse wave velocity (AoPWV) is supposed to be the “gold standard” for estimating aortic stiffness as an independent variable for foreseeing adverse cardiovascular events due to its responsibility for the majority of pathophysiological pathways that results in causing cardiovascular outcomes [9,10]. Brandt MC et al. highlighted the relationship linking RDN and arterial wall stiffness as well as central haemodynamics [11]. These findings ignited new academic discussions and scientific research about a new potential additive value of RDN procedure apart from decreased arterial blood pressure.

In our previous study, we also observed a significant reduction in blood pressure and AoPWV that was sustained for up to 12 months after the RDN procedure [12].

As there is a lack of studies that investigated long-term changes after RDN, we aimed to investigate blood pressure changes and aortic stiffness 48 months after the procedure. In the past few years, many researchers frankly recognise the fact that polypharmacy (defined as using more than five medications [13]) and drug non-adherence are among the most frequent causes of RH [14]. In light of this knowledge, we also aimed to retrospectively analyse the influence of the number of medications on changes in blood pressure and AoPWV.

## 2. Materials and Methods

### 2.1. Study Design

A prospective single arm interventional study was conducted between March 2012 and December 2016, and a total of 243 patients with suspected resistant hypertension were referred to university hospital hypertension specialist. They went through a detailed examination according to local hospital protocol, which contained magnetic resonance tomography (MRT) of whole aorta, renal arteries and adrenal glands to rule out secondary hypertension [12]. If MRT was unable to be performed, a computed tomography scan was conducted. Blood analysis contained aldosterone, renin (aldosterone to renin ratio calculated), metanephrine and normetanephrine. Concomitantly, at the time of first patient examination, the patient’s antihypertensive treatment regimen was checked and corrections were made if necessary. One month after the antihypertensive treatment modification, office and 24 h ambulatory blood pressure measurements were accomplished. The 24 h BP measurements were accepted for further analysis if at least 21 daytime readings and 12 night-time readings had been recorded. Daytime was specified as time between 7:00 a.m. and 10:00 p.m. and night-time was defined as time between 10:00 p.m. and 7:00 a.m. For the patients for whom we failed to achieve target office or ambulatory BP (after 1 month), we recruited them in the study. After exclusion secondary hypertension causes and confirming treatment RH, only 81 patients were left: eight patients refused the procedure and 73 had undergone RDN. The study was conducted in accordance with the Declaration of Helsinki, and the protocol was approved by the Vilnius Regional Biomedical Research Ethics Committee (No. 158200-13-641-205 and 158200-18/3-1011-511). All patients signed an informed consent form prior to formal enrolment in the study.

### 2.2. Inclusion Criteria

Consecutive adult patients (>18 years old) treated for resistant hypertension with anatomical eligibility of renal artery for treatment (renal arteries >3 mm in diameter and >20 mm in length, without significant renal artery atherosclerosis, abnormality or stenosis and history of previous renal artery stenting) were included in the study.

### 2.3. Exclusion Criteria

Patients with acute myocardial infarction, unstable angina pectoris, cerebrovascular accident within the last 6 months or haemodynamically significant valvular disease or chronic kidney disease (CKD) stage 4 or higher (according to KDIGO 2012 guidelines [15]) were excluded from the study. Patients with secondary causes of hypertension (such as severe sleep apnoea (apnoea–hypopnea index > 30 events per hour), pheochromacytoma) were also excluded from the study and referred to other specialists.

### 2.4. Data Collection

Patients’ comorbidities, medication regimen, clinical signs, laboratory parameters, office and 24 h ambulatory BP and aortic pulse wave velocity measurements were obtained according to the local protocol.

### 2.5. Renal Artery Sympathetic Denervation

Renal artery denervation was performed using either Symplicity FlexTM catheter powered by the Symplicity G2TM generator, or Symplicity SpyralTM catheter powered by the Symplicity G3TM generator (Medtronic Plc, Galway, Ireland).

After gaining arterial access, F-6 to 8-F hydrophilic sheaths were used and 5000 heparin units were administered to manage anticoagulation. After cannulating renal artery ostia using a standard guiding catheter, angiography of the renal arteries was performed. A renal denervation catheter was then positioned distally in the renal artery so to come into contact with the inner wall of the artery distally. When we used the Symplicity FlexTM catheter (singlepoint 2 minute ablation), after each ablation, the catheter was rotated 90 degrees and slightly pulled back.

When we used Symplicity SpyralTM catheter (four-point 1 minute simultaneous ablations), the methodology of the procedure was slightly different. First, a 0.014-inch wire was directed through the guiding catheter to the renal artery and a denervation catheter was placed on the wire, pushing four sequential electrodes. Once the wire is withdrawn from the area of electrodes, the ablation catheter takes a spiral shape and sticks to the inner wall of the artery. After having controlled that there was a good contact between the electrodes and arterial wall (i.e., no marked fluctuations of the impedance trace), radiofrequency ablation was then performed and the procedure was continued in accordance with the methodology above. 

If angiography showed accessory renal arteries of ≥3 mm in diameter, the radiofrequency ablation procedure was then repeated in these arteries following the same methodology. The number of RF ablations depended on the vascular anatomy. After the radiofrequency applications, control angiography was performed to exclude complications.

After the procedure, all patients received antiaggregant therapy with aspirin or clopidogrel for at least 1 month.

### 2.6. Arterial Stiffness and Wave Reflection Measurements

The measurement and processing technique is described in detail in our group’s previous publication [12]. Briefly, the parameters of arterial stiffness and wave reflection were estimated by applanation tonometry and analysed according to European expert consensus (SphygmoCor v.8.0; AtCor Medical, Sydney, Australia) [9].

### 2.7. Follow-Up

Patient follow-up was performed from April 2012 until December 2019. Arterial wall stiffness and central haemodynamics were obtained before the procedure and at 3, 6, 12, 24 and 48 months after the RDN procedure. Adding to this, office and 24 h ambulatory BP measurements were performed at the same time points. During each visit, compliance with medical treatment was checked in accordance with the patient’s medication passport and electronic health records. At the beginning of the study our focus was to continue stable antihypertensive treatment during the entire observation period. At the moment of clinical evaluation, all patients had a stable cardiac status; there were no significant changes in drug regimen from baseline up to 24 months. During next 24 months, the patients were mainly observed and treated by their family physicians and cardiologists. The final 48-month examination was again performed at the university hospital Centre for Resistant Hypertension. Five patients died during the follow-up period and 15 patients were lost to follow-up, as they refused or were unable to proceed to final follow-up at 48 months. This was largely influenced by global the COVID-19 pandemic, as we were not able to add research data in 2020.

### 2.8. Statistical Analysis

Statistical analysis was accomplished using the following software: R statistical software package V 4.0.2 (© The R Foundation for Statistical Computing), Rstudio Version 1.3.959 © 2021–2020 RStudio, PBC, IBM SPSS Statistics V.23, G*Power V. 3.1.9.4 Universität Düsseldorf, Germany.

Interval and ratio variables were described by minimum (Min) and maximum (Max) values, means and standard deviations (SD), medians, first quartiles (Q1), third quartiles (Q3) and interquartile deviations (IQR 75%). Shapiro–Wilk and Kolmogorov–Smirnov (K–S) tests were used to check the data for normality. Ordinal and nominal variables were characterised by frequencies and percentages across the corresponding subset of the sample.

In order to assess a statistically significant influence of relevant independent variables on the dependent variable, we created models based on linear regression equations. In order to test hypotheses about the equality of population means, we used the *t*-test and ANOVA test.

In order to evaluate a statistically significant relationship between the ordinal and nominal variables, we used the Chi-Square Test of Independence. To assess the statistically significant difference among the dependent groups, we used the Friedman rank sum test (or simply Friedman test). We used the Dunn–Conover test for pairwise multiple comparisons of the ranked data.

To measure the effect size between variables, we used Kendall’s coefficient of concordance (Kendall’s W), a strength-of-relationship index. Kendall’s W uses Cohen’s interpretation guidelines of 0.1 ≤ 0.3 (small effect), 0.3 ≤ 0.5 (moderate effect) and ≥0.5 (large effect).

Relationships between variables were considered statistically significant when the *p*-value was less than 0.05 (*p* < 0.05) and a statistical test power of 1-ß was equal to 0.95 (1-ß = 0.95).

## 3. Results

### 3.1. Study Population

The study included 73 patients with RH who underwent bilateral RDN procedure. After approximately 48 months of follow-up, five patients died (6.8%); two deaths were related to cardiovascular causes, but none of them directly to the renal denervation procedure. A total of 15 patients (20.6%) were lost to follow-up or were not able to perform all tests required in the study protocol, 4 patients’ (5.5%) data were not included in the analysis due to permanent atrial fibrillation and 49 patients (67.1%) remained in the final analysis.

Mean patient age was 56 [±7.76] years. Pre-intervention median (IQR) office blood pressure was 180(40)/110(15) mmHg in the study group of patients treated with an average of 6.25 [5.8–6.9] antihypertensive drugs at maximum or maximum tolerated doses. Baseline characteristics of the patients before the procedure are presented in Table 1.

### 3.2. Time Course of Change in Blood Pressure

**Figure 1:**
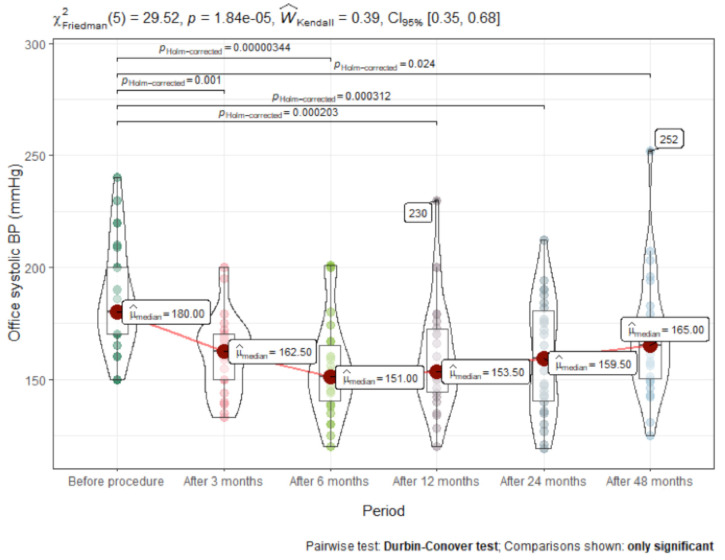
Office systolic blood pressure(BP) changes after renal artery denervation (RDN) (mmHg).

When analysing our follow-up data, we observed a significant decrease in the median office BP from baseline at month 3, i.e., from 180/110 to 162.5/94.5 mm Hg, which was sustained at months 6, 12, 24 and 48 (Figure 1). Immediately after the procedure, office blood pressure dropped significantly, then tended to become higher over time, but never reached the pre-procedure value.

**Figure 2:**
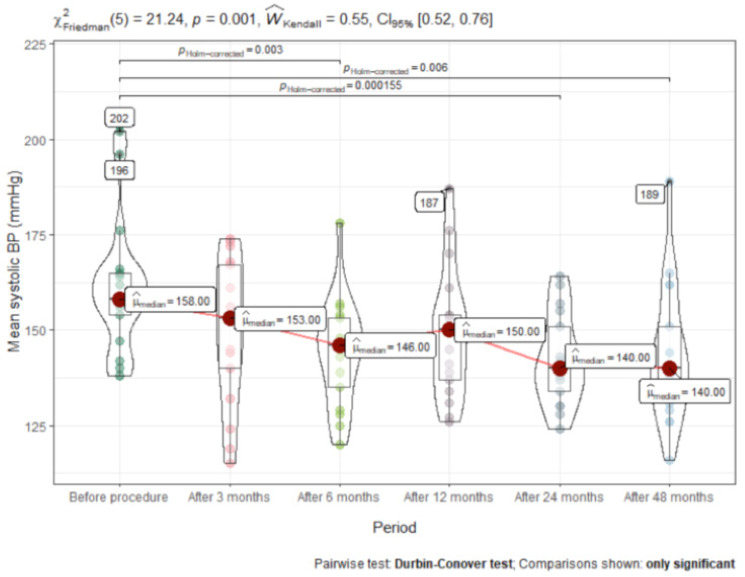
Mean systolic 24 h ambulatory BP after RDN (mmHg).

**Figure 3:**
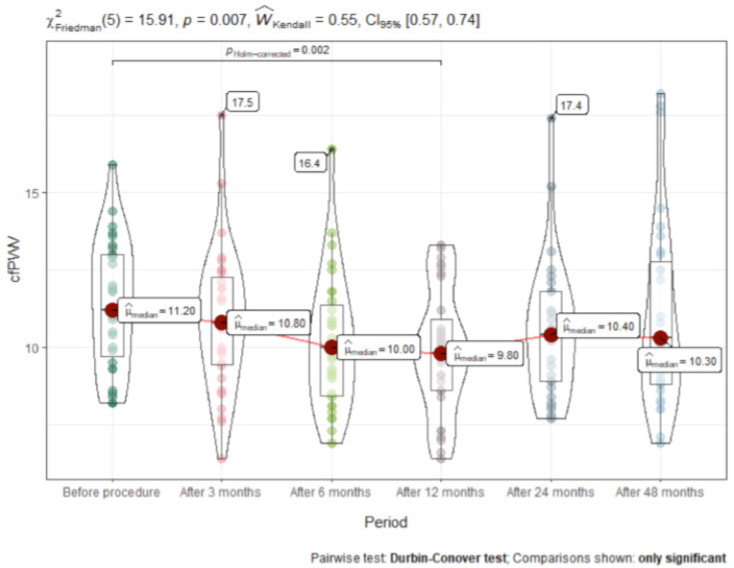
Carotid-femoral pulse wave velocity (CfPWV) dynamics after RDN procedure (m/s).

A significant decrease in the median 24 h ambulatory BP from baseline was measured at month 6, i.e., from 158/100 to 146/89 mmHg, and persisted at months 12, 24 and 48. Office and 24 h ambulatory BP values with regard to other timeframes are presented in Figure 2 and Figure 3 and Table 2. After 48 months, mean reduction in 24 h ambulatory systolic BP was (mean difference [MD] −11 ± 25mm Hg; (95% CI, −20 to −2; *p* < 0.001), while office systolic BP reduced by −7 ± 23 mm Hg; (95% CI, −24 to −1; *p* < 0.02).

### 3.3. Time Course of Change in Carotid-Femoral Pulse Wave Velocity (cfPWV)

We observed a significant decrease in median [IQR] cfPWV 12 months after the procedure (drop from baseline 11.2 [3.15] m/s (95% CI 6.1 to 16.2) to 9.8 [2.1] m/s (95% CI 6.1 to 13.7; *p* = 0.002), mean reduction −1.4 ± 0.98 m/s (95% CI −3.1 to −0.3). cfPWV remained unchanged at 6 and 24 months and was 10.3 [4.0] m/s (95% CI 6.9 to 17.8) at the 48-month follow-up. (Figure 3).

**Figure 4:**
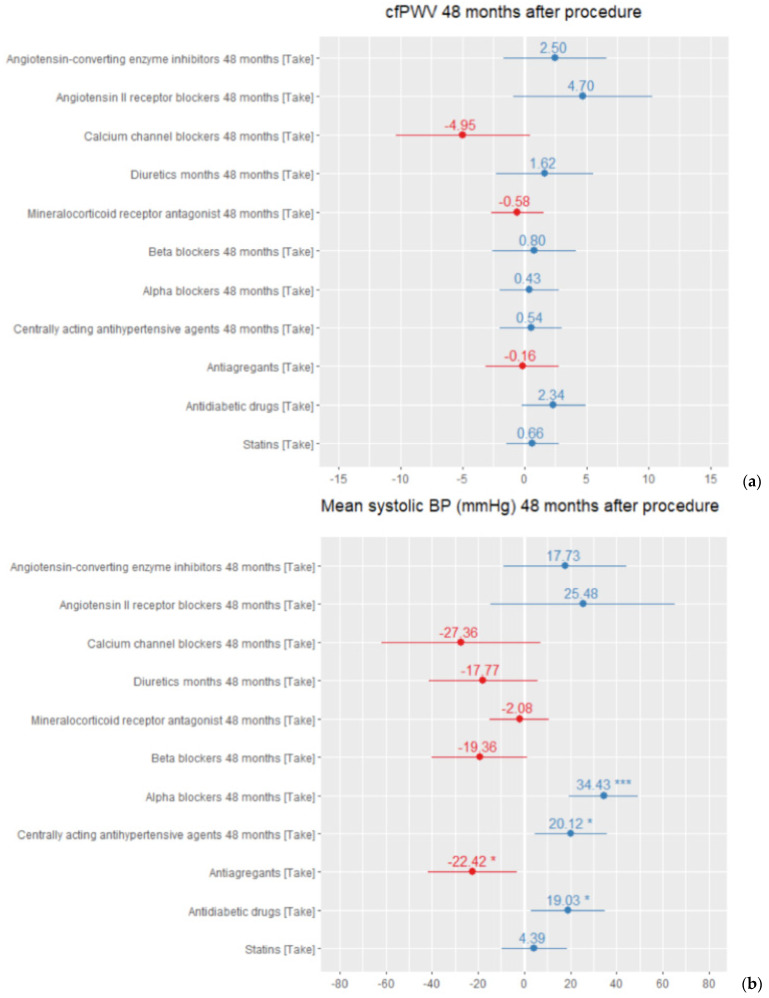
Mean regression model for drug treatment influence on (**a**) arterial stiffness (CfpWV in m/s) after 48 months (*R^2^* = 0.66, adjusted *R^2^* = 0.44, *p* = 0.01). (**b**) Office systolic blood pressure (in mmHg) after 48 months (*R^2^* = 0.66, adjusted *R^2^* = 0.44, *p* = 0.01). * *p* < 0.05, *** *p* < 0.001.

The regression analysis showed that centrally acting antihypertensive agents and alpha-blocker usage in treatment schemes had presented significantly worse results in blood pressure control. We found no significant influence of different drug classes on arterial stiffness during the follow-up period (Figure 4). The total number of antihypertensive medication remained the same after 48 months 5.97 ± 1.1 versus 5.45 ± 2.2 at the beginning. The higher the total number of pills taken by the patient, the higher their office blood pressure measurements.

**Figure 5:**
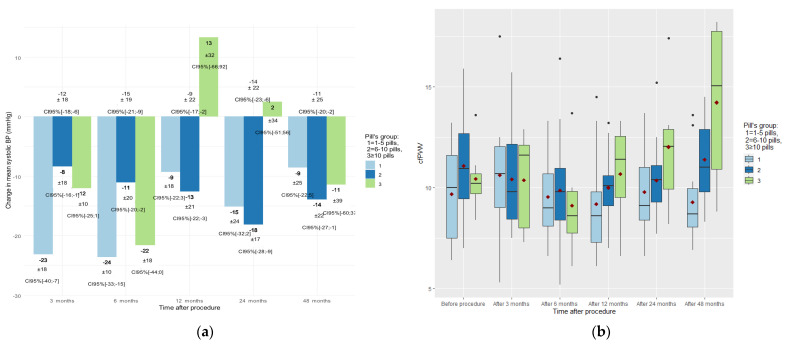
Influence of the total number of pills on (**a**) 24 h mean systolic blood pressure mean reduction (in mmHg) and (**b**) arterial stiffness, CfPWV (in m/s) over observation period.

We divided the groups by the number of pills taken (group 1: 1–5 pills; group 2: 6–10 pills; group 3: >10 pills) and compared them in time with blood pressure and cfPWV. There were no significant differences found between pill groups in 24 h BP monitoring (Figure 5a). However, we found significant differences in the mean cfPWV between the groups after 48 months (group 1: 8.1 ± 1.6 m/s (95% CI 6.8 to 10.3); group 2: 10.9 ± 1.8 m/s (95% CI 8.4 to 14.8); group 3: 15.1 ± 2.6 m/s (95% CI 8.7 to 17.8)) (*p* = 0.003) (Figure 5b).

## 4. Discussion

To our knowledge, our study has shown, for the first time, that the antihypertensive effect lasts up to 48 months after RDN, and our data expand beyond the previously reported 36-month RDN sustainability period [16]. Since the mean prescribed number of antihypertensive drugs remained the same, this suggests that the procedure has a positive antihypertensive effect. However, we did not manage to reduce patients’ blood pressure to optimal values. These difficulties, in our opinion, were related to polypharmacy (>6 pills) and the high total number of pills (*n* = 7.33 (±2.44) prescribed for treating arterial hypertension and concomitant pathologies. For instance, some of our patients were taking as many as 14 pills per day. As presented in Figure 5, polypharmacy had a negative tendency short- and long-term effect on patient blood pressure and arterial stiffness after RDN. We hypothesise that antihypertensive treatment variability in group 3 (>10 pills) is related to their more difficult-to-control HTN and higher number of comorbidities, placing them in a higher cardiovascular risk group (Figure 5a). This is why we presume that the high average number of antihypertensive medications (5.1) in Simplicity HTN-3, as compared to the significantly lower number (2.2) in Spyral ON-MED or no medication as in Spyral OFF-MED and Radiance-HTN SOLO trials, might be one of the reasons for the Simplicity HTN-3 failure [17]. The effectiveness of the RDN procedure in blood pressure reduction has remained a topic for research and discussion over the past decade. In the recent meta-analysis by Cheng, X [18], 12 randomised controlled trials with a total of 1539 individuals were analysed. The conclusion of their analysis was that RDN procedure resulted significant and clinically relevant decreases in 24 h ambulatory BP and office BP (−8.93/−4.49 mm Hg) in poorly controlled HTN. The extent of antihypertensive effect could foresee the progression of major cardiovascular events and deaths [18].

Arterial wall stiffness is recognized as an important independent risk factor for cardiovascular outcomes causing increased systolic BP and increased pulse pressure in the microcirculation of target organs resulting in their damage. Such conditions as ageing, hypertension, diabetes mellitus and CKD increase arterial wall stiffness. This process is decided by common molecular and cellular pathophysiological pathways, resulting in endothelial dysfunction [19,20]. Despite still unknown mechanisms of RDN, there are a number of papers confirming a beneficial impact of the procedure on arterial wall stiffness and central haemodynamics [11]. Recent research in the field of cell biology, with a focus on nuclear mechanotransduction, mitochondrial oxidative stress, metabolic disorders, genetics and epigenetics, will provide us with knowledge about targeting different molecular pathways, at different times of exposure to risk factors, that will result in arterial wall de-stiffening without affecting artery function [21].

According to a study by Diaz et al. [22], a mean breakpoint in vascular ageing is 50 years, and this is even faster in hypertensive patients. According to their data, CfPWV increases over time in patients with hypertension were substantially greater when compared to normotensive patients. In the study by Diaz et al., baseline CfPWV in hypertensive patients was 8.04 ± 1.8 m/s, while in our group it was much higher, at 10.77 ± 2.9 m/s, as could be expected in resistant hypertension population. Moreover, our follow-up results at 12, 24 and 48 months presented a persistent effect of RDN on maintaining arterial stiffness at decent levels. These findings supports the results of a comparable study by Ott et al. [23] and allows us to summarize that longer beneficial impact on arterial wall stiffness may trigger a greater cardiovascular disease risk and mortality reduction. As trials of RDN effects on hard end points are still underway and results are not yet available, the meaning of intermediate end points such as CfPWV is of considerable importance.

From the perspective of personalised HTN treatment strategy, despite a number of trials and meta-analyses, there still are many unknown facts related to the RDN procedure. Several studies report that younger patients suffering from abdominal obesity, combined systolic–diastolic hypertension after exclusion of secondary causes and high baseline heart rate would benefit most from the RDN procedure [17]. In our study, we also observed that polypharmacy had an association with worse long-term blood pressure control and arterial stiffness. We presume that patients whose medication regimen included central acting agents, alpha blockers and antidiabetic drugs had refractory hypertension, as the linear regression model demonstrated a statistically significant influence on worse BP results in such patients. We also observed a phenomenon that CfPWV was slightly higher in patients treated for diabetes mellitus than in patients treated for other comorbidities, such as dyslipidaemia or coronary artery disease. However, due to our small cohort, we did not make any further assumptions about comorbidities’ role in these hypertensive patients’ treatment results. Likewise, patients whose treatment regimen included ACEi and ARB had slightly higher BP and CfPWV values, but not significantly. This may be explained by the fact that 95.9% our patients received ACEi or ARB and the mean target BP was not reached (<140/90 mmHg). This is why the regression model depicted these main drug classes in a negative shade. However, the small sample size limits our ability to draw a clear conclusion about such data.

Trials by our colleagues analysed above and our data suggest that renal denervation could be an effective adjuvant procedure to optimised medication regimens in treating resistant or refractory hypertension.

## 5. Conclusions

The blood pressure lowering effect lasts up to 48 months after RDN, with no worsening of arterial stiffness compared to baseline. In our study, polypharmacy was associated with increased CfPWV at 48 months after RDN.

### Limitations

The major limitation of the present research is the size of our study group and our lack of a control group. Therefore, the study is not a comparative study, but rather an interventional study that represents a trend of blood pressure and arterial wall stiffness after RDN. Another drawback is the deficiency of direct data measuring sympathetic nervous activity. The third limitation is the lack of patients’ plasma or urine drug concentration analysis, given that drug non-adherence has been a hot topic in the past decade. Finally, we lost some follow-up data due to the outbreak of the COVID-19 pandemic.

## Figures and Tables

**Table 1:** Baseline characteristics of the study population before procedure.

Variables	Values (Mean (SD) [Min, Max])	
Age, years	56.0 (7.76) [33.0, 72.0]	
Sex		
Male	*n* = 34 (46.5%)	
Female	*n* = 39 (53.5%)	
Waist circumference, cm	115.5 (10.7) [95, 129]	
Height, cm	168.6 (8.6) [150, 186]	
Weight, kg	97.6 (17.7) [60.0, 134]	
BMI (kg/m^2^)	34.3 (5.6) [22.1, 51.2]	
Drug regimen	**Before procedure**	**48 months after procedure**
Number of antihypertensive drugs	5.97 (1.1) [4–11]	5.45 (2.2) [3–11]
Total number of pills	7.33 (2.44) [2–14]	6.92 (3.37) [2–16]
Prescribed drug classes		
ACE-I/ARB	73 (100%)	47 (95.9%)
Diuretics	67 (91.8%)	39 (79.6%)
Calcium channel blockers	63 (86.3%)	42 (85.7%)
Beta blockers	66 (90.4%)	27 (55.1%)
Mineral receptor antagonists	48 (65.7%)	43 (87.8%)
Centrally acting agents	57 (78.1%)	24 (49.0%)
Alpha blockers	45 (61.6%)	32 (65.3%)
Oral antidiabetic drugs *	29 (39.7%)	18 (36,7%)
Oral anticoagulants	5 (6.8%)	8 (16.3%)
Antiplatelet drugs	21 (28.7%)	23 (46.9%)
Statins	32 (44.4%)	24 (48%)

Values are expressed as *n*, median (interquartile range), mean (SD) [min;max] or *n* (%), unless otherwise stated. ACE: angiotensin-converting enzyme; ARB: angiotensin-receptor blocker; BMI: body mass index; *n*: number of subjects with available data. * Biguanides, sulphonylureas.

**Table 2:** Main blood pressure parameters over observation period.

Variable (Median IQR)	Before Procedure	After 3 months	After 6 months	After 12 months	After 24 months	After 48 months	*p*-Value	Effect Size
Office systolic BP	180 (40.0)	162.5 (26.2)	151 (29.0)	153 (32.0)	169 (38.5)	165 (34.0)	<0.001	0.17
Office diastolic BP	110 (15.0)	94.5 (16.2)	89.5 (18.5)	93.5 (17.0)	95 (21.5)	95 (16.5)	<0.001	0.12
Office heart rate	68 (8.2)	64 (10.0)	69 (10.5)	72 (15.0)	63 (22.5)	74 (14.8)	0.052	0.03
Mean systolic BP	158 (23.5)	153 (31.0)	146 (26.0)	150 (25.0)	140.5 (20.8)	140 (26.5)	<0.001	0.07
Mean diastolic BP	100 (14.2)	92 (19.0)	89 (13.0)	92 (15.2)	90.5 (14.8)	86 (16.2)	0.01	0.04
Mean heart rate	71.5 (12.8)	68 (11.0)	67 (14.5)	66 (11.0)	65.5 (13.5)	70 (11.5)	NS	0.01
Daytime mean systolic BP	160 (23.5)	153 (28.0)	150 (26.0)	154 (27.8)	145 (18.8)	143 (22.8)	<0.001	0.08
Daytime mean diastolic BP	104 (16.5)	94 (18.0)	94 (13.5)	93 (18.0)	92 (15.0)	98 (14.8)	0.001	0.05
Daytime mean heart rate	75.0 (13.0)	70.5 (11.2)	71.0 (15.0)	70.0 (12.5)	70.0 (14.5)	71.5 (14.5)	NS	0.01
Night-time mean systolic BP	154 (29.0)	141 (25.0)	131 (28.8)	141 (27.0)	130 (37.2)	133 (40.2)	0.014	0.05
Night-time mean diastolic BP	91 (17.5)	84 (19.0)	82 (12.8)	84 (15.2)	81 (18.0)	77 (17.0)	NS	0.02
Night-time mean heart rate	64.0 (11.8)	62.0 (11.2)	60.0 (9.8)	60.0 (8.5)	61.0 (14.8)	61.5 (13.2)	NS	0.01
Systolic BP dipping	7.1 (13.0)	5.5 (10.3)	7.1 (10.3)	7.5 (7.3)	6.4 (11.6)	8.6 (13.0)	NS	0.00
Diastolic BP dipping	10.2 (11.9)	9.6 (12.4)	11.9 (8.5)	11.7 (9.7)	8.4 (8.8)	11.6 (11.9)	NS	0.02

IQR: interequartile range; BP: blood pressure; NS: not significant.

## Data Availability

The data presented in this study are available on request from the corresponding author. The data are not publicly available due to patient privacy.
